# Prolonged linezolid therapy induces progressive mitochondrial dysfunction in human peripheral blood mononuclear cells

**DOI:** 10.1186/s12967-026-08406-5

**Published:** 2026-06-09

**Authors:** Marta Martínez-Guitián, Francisco Cajade-Pascual, Diana Carolina Castro-Fernández, Andrea Cuartero-Martínez, Rubén Nogueiras, Iván Fernández-Castro, Sonia Molinos-Castro, Ignacio Ortea, Irene Zarra-Ferro, Miguel González-Barcia, Anxo Fernández-Ferreiro, Cristina Mondelo-García

**Affiliations:** 1 FarmaCHUSLab, Health Research Institute of Santiago de Compostela (IDIS), Santiago de Compostela, 15706 Spain; 2 Pharmacy Department, University Clinical Hospital of Santiago de Compostela (SERGAS), Santiago de Compostela, 15706 Spain; 3 CIMUS, University of Santiago de Compostela- Health Research Institute of Santiago de Compostela, Santiago de Compostela, Galician, 15782 Spain; 4 Galician Agency of Innovation (GAIN), Xunta de Galicia, Santiago de Compostela, Spain; 5 Internal Medicine Department, University Clinical Hospital of Santiago de Compostela (SERGAS), Santiago de Compostela, 15706 Spain; 6 Proteomic Department, Center for Research in Nanomaterials and Nanotechnology (CINN-CSIC), Health Research Institute of Principado de Asturias (ISPA), Oviedo, Spain

**Keywords:** Linezolid, Mitochondrial dysfunction, Proteomic profile, Complex I, Toxicity

## Abstract

**Background:**

Linezolid commonly causes hematologic toxicity, especially thrombocytopenia, during extended treatment. This effect is linked to the inhibition of mitochondrial protein synthesis, which impairs oxidative phosphorylation and cellular energy production. Although reduced complex IV activity has been observed in long-term therapy, the connection between treatment duration and mitochondrial dysfunction in humans remains insufficiently defined.

**Methods:**

Forty patients were included and stratified by treatment duration: 2–7 days (group 1), 8–14 days (group 2) and more than 14 days (group 3). To evaluate mitochondrial function in peripheral blood mononuclear cells (PBMCs) from patients treated with linezolid for different durations, Seahorse XF was used. To assess protein expression changes associated with mitochondrial toxicity, LC-MS/MS was used. Differentially expressed proteins were identified in R based on fold-change thresholds (|log₂FC|≥0.58) and *p* < 0.05, and results were visualized with volcano plots and STRING interaction networks.

**Results:**

All the groups were similar in terms of variables and characteristics. Mitochondrial respiration declined progressively with treatment duration and platelet counts showed a parallel reduction. Proteomic analysis identified one cluster of downregulated protein in group 2 of patients and two in group 3; they were involved in mitochondrial ATP synthesis—mainly subunits of complexes I and IV of the respiratory chain—supporting a concordant functional and molecular pattern of mitochondrial impairment.

**Conclusions:**

Prolonged linezolid treatment was associated with progressive mitochondrial dysfunction, as reflected by both functional assays and proteomic profiles. Concordant changes in respiration and protein expression support mitochondrial involvement in linezolid-related toxicity and suggest that, in addition to previously reported alterations in complex IV, complex I proteins may also be affected in treated patients.

**Supplementary Information:**

The online version contains supplementary material available at 10.1186/s12967-026-08406-5.

## Graphical Abstract

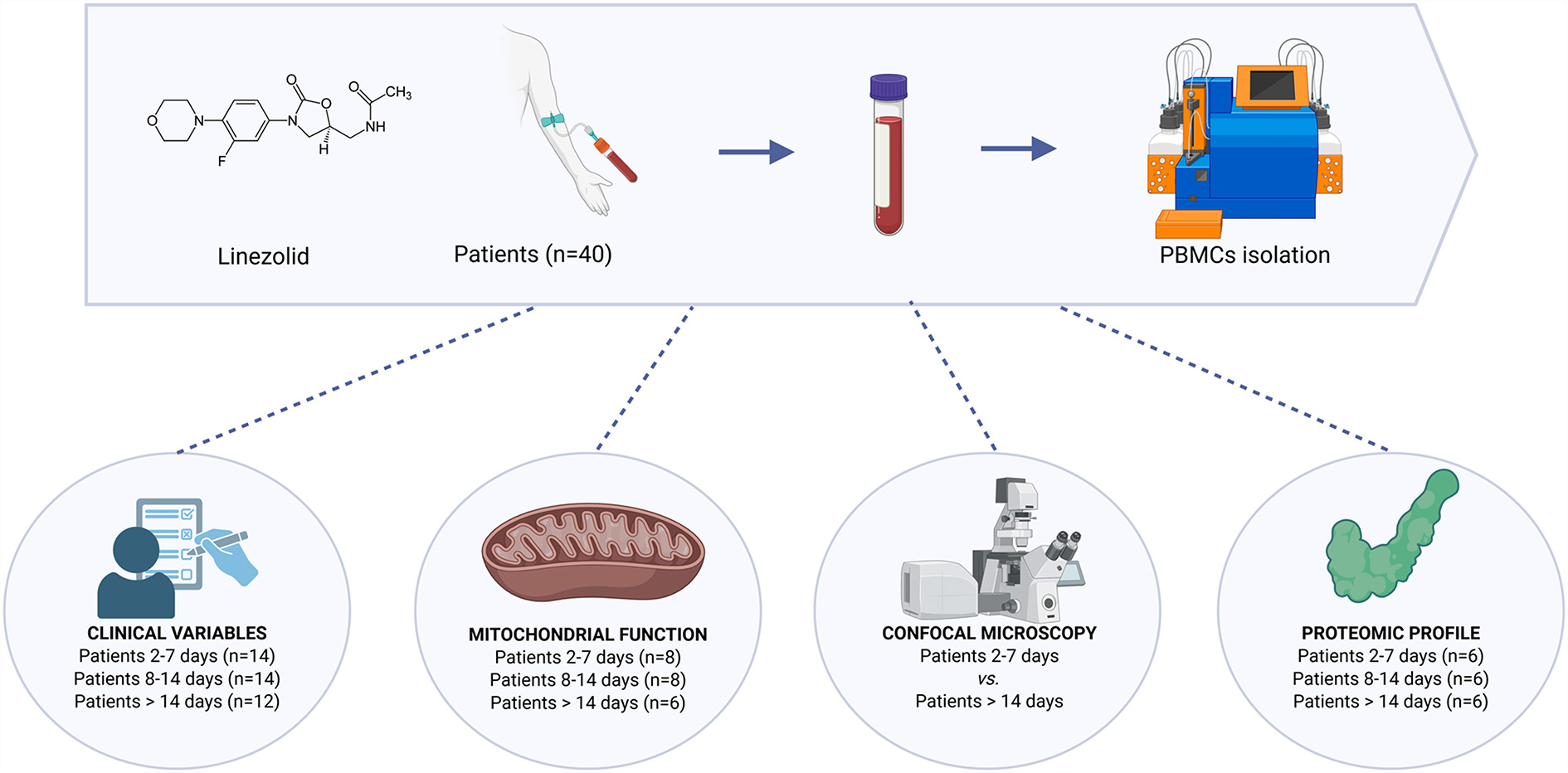

## Background

Linezolid is an oxazolidinone-class antibiotic used to treat severe infections caused by resistant Gram-positive bacteria, including methicillin-resistant *Staphylococcus aureus*, penicillin-resistant *Streptococcus* species, and vancomycin-resistant *Enterococcus*. Linezolid binds to the 23S rRNA of the large subunit in the prokaryotic ribosome, thereby blocking the attachment of aminoacyl-tRNA and inhibiting bacterial protein synthesis [1].

Clinical studies have shown that thrombocytopenia is among the most frequent adverse effects associated with linezolid therapy. In a review by Zou et al., 1,139 cases of thrombocytopenia and 549 cases of decreased platelet count were reported from U.S. Food and Drug Administration (FDA) Adverse Event Reporting System database [2]. Moreover, linezolid-induced hematologic toxicity, particularly thrombocytopenia and anaemia, has been observed to increase with the duration of treatment. These effects are linked to inhibition of mitochondrial protein synthesis and subsequent impairment of oxidative phosphorylation (OXPHOS) in blood cells [3, 4]. In addition to haematological effects, other adverse reactions have also been reported, including hyperlactatemia as well as lactic and metabolic acidosis. These adverse effects are also observed in patients undergoing prolonged treatments. Indeed, both the FDA and the European Medicines Agency advise against the use of linezolid for periods exceeding 28 consecutive days. Nonetheless, in clinical practice, adherence to this recommendation is not always attainable [1, 5], as certain infections require treatment courses of at least four to twelve weeks [6, 7]. In cases of drug-resistant tuberculosis, treatment may be prolonged for as long as 8 months, although adverse effects often begin to emerge after approximately 60 days [8]. Management of nocardiosis can demand even longer courses, sometimes extending to twelve months [9]. Nevertheless, linezolid remains an excellent option for outpatient therapy due to its high bioavailability and favourable tissue penetration [10].

Most adverse events linked to linezolid are believed to arise from its interference with mitochondrial function. Because mitochondrial ribosomes share structural similarities with bacterial ribosomes, they are susceptible to interference by ribosome-targeting antibiotics such as linezolid. This inhibition compromises oxidative phosphorylation (OXPHOS), particularly affecting complexes I and IV of the electron transport chain, which results in reduced ATP production and cellular energy failure [5, 11]. Specifically, a reduction in the activity of complex IV (cytochrome c oxidase) has been observed in patients undergoing prolonged treatment, as it is partially synthesized by mitochondrial ribosomes, unlike complex II which is synthesized in the cytoplasm [12]. In vitro investigations indicate that mitochondrial function may be reduced by 50–75% in basal and spare respiratory capacity [13, 14]. Nonetheless, these findings are based on limited patient cohorts, and comprehensive clinical confirmation remains pending [12, 15–17].

Proteomics is the large-scale study of proteins in cells, tissues, or organisms, offering a powerful way to discover new biomarkers for diagnosing and characterizing disease [18]. Mass spectrometry–based proteomics measures peptide masses and fragmentation patterns with high specificity, avoiding limitations like cross-reactivity seen in traditional assays [19]. In turn, pharmacoproteomics extends this approach to drug research by examining how medications alter protein expression, helping identify protein signatures linked to mitochondrial dysfunction and toxicity, such as those that may occur during linezolid treatment.

Peripheral blood mononuclear cells (PBMCs) are considered a relevant biological model because of their established role as a surrogate for systemic mitochondrial status [20]. These cells exhibit high aerobic metabolic activity and are accessible through routine blood draws, which offers a significant advantage over invasive techniques. Also, they have been validated as a proxy for mitochondrial dysfunction in studies of mitochondrial toxicity from pharmacological agents and in primary mitochondrial disorders [5, 15]. In addition, their experimental versatility allows for detailed proteomic profiling, positioning them as a valuable source of biomarkers for therapeutic response.

This study represents the first effort to evaluate both mitochondrial function and the proteome of PBMCs of patients undergoing linezolid treatment at different time points. Its primary objective is to characterize the time-dependent relationship between linezolid exposure and mitochondrial dysfunction, and to identify the protein-level correlates of this association. To our knowledge, this is the first clinical proteomic study to identify downregulation of complex I subunit proteins in patients receiving linezolid therapy, extending prior in vitro and animal evidence to the human clinical setting.

## Methods

### Design of the study

This was a prospective observational study conducted at a tertiary hospital (University Hospital Complex of Santiago de Compostela) with ethics approval from the Galician Network of Ethics Committees (Ethical Approval Code: 2023/048). The study followed Good Clinical Practice and the Declaration of Helsinki, and all participants provided written informed consent. Adults over 18 years treated with linezolid for at least two days were included, while patients without consent, with linezolid allergy, or who were pregnant, or breastfeeding were excluded.

Patients were selected from three-time ranges according to linezolid treatment duration at the time of blood sampling: 2 to 7 days (group 1), 8 to 14 days (group 2), and more than 14 days (group 3).

Demographic, clinical, anthropometric, and analytical data were collected, including age, sex, type of infection, comorbidities, BMI, obesity status, concomitant treatments affecting mitochondrial function and haematological and inflammatory markers. Haematological variables were recorded at treatment initiation and at sample collection. Thrombocytopenia was defined as a ≥ 25% decrease in platelet count from baseline or a final count below 100 × 10³/µL [21]. These variables were chosen due to their potential influence on linezolid-associated adverse effects.

### Sample collection and isolation of PBMCs

Peripheral blood (12 mL) was collected at trough concentration into BD Vacutainer^®^ K2E (EDTA) 7.2 mg™ tubes (Becton Dickinson, USA) and stored at room temperature, protected from light, for up to 48 h before processing. Each patient was sampled at a single time point during their treatment course. PBMCs were isolated using an automated magnetic separation system (AutoMACS Pro Separator, Miltenyi-Biotec S.L., Spain) with sedimentation and negative selection protocols: Sedimentation Kit II, human and StraightFrom^®^ Whole Blood PBMC Isolation Kit (both Miltenyi-Biotec S.L., Spain). Isolated PBMCs were cryopreserved in liquid nitrogen using a standardized freezing solution until analysis.

### Evaluation of mitochondrial respiratory function

Mitochondrial function was assessed by measuring oxygen consumption rate (OCR) using the Seahorse XF Analyzer (Agilent, USA). Experimental groups (2 and 3, both *n* = 8) were compared with a control group (1, *n* = 8), with 10⁶ cells seeded per well on coated plates. The Cell Mito Stress Test (Agilent, USA) was performed under standardized assay conditions to measure basal respiratory capacity (BRC), proton leak after oligomycin, maximal respiratory capacity (MRC) following carbonyl cyanide p-trifluoromethoxyphenylhydrazone (FCCP), and non-mitochondrial respiration after rotenone/antimycin A. OCR values were normalized to cell number for analysis.

### Confocal microscopy

PBMCs isolated from group 1 and group 3 were compared. 2.5 × 10^6^ cells were cultured in serum-free medium, fixed after 3 h, and imaged by confocal microscopy (Leica TCS SP8 microscope). Mitochondrial reactive oxygen species were detected using the MitoSOX™ Red kit (Thermo-Fisher, USA) with nuclei counterstained with DAPI. Imaging was performed under defined excitation and emission settings, and experiments were conducted in triplicate. These analyses are exploratory and qualitative observations complementary to the functional data.

### Pharmacoproteomic characterization of mitochondrial alterations

Experimental groups were compared with the group 1 (*n* = 6) and included group 2 (*n* = 6), and group 3 (*n* = 6). The iST Kit (PreOmics, Germany) was used for peptide preparation for proteomic analysis following the manufacturer’s instructions. The samples were subsequently vacuum-dried and stored at − 80 °C until liquid chromatography–mass spectrometry (LC–MS/MS) analysis.

Peptide samples (200 ng per injection) were analysed by LC–MS using an Orbitrap Exploris 480 mass spectrometer coupled to a Vanquish Neo HPLC system (both Thermo-Fisher Scientific, USA). Peptides were separated in trap-and-elute mode on a PepMap trap cartridge and an Aurora analytical column using a 46-min nanoLC gradient at 250 nL/min with water/acetonitrile containing 0.1% formic acid. The LC gradient was programmed as follows: 4–25% B over 30 min, 25–40% B over 5 min, 40–85% B over 5 min, followed by a column wash at 85% B for 4 min, yielding a total run time of 4 min.

Data were acquired using a data-independent acquisition (DIA) strategy consisting of one full MS scan (400–900 m/z, 60,000 resolution) followed by 42 MS/MS scans with 12 m/z isolation windows.

To assess reproducibility, one randomly selected sample was analysed in triplicate, and coefficients of variation (CVs) were calculated for each identified protein.

### Bioinformatic and statistical analysis

Mitochondrial function data were analysed by ANOVA with Dunnett’s post hoc multiple comparisons test. Statistical significance was set at *p* < 0.05. Each measurement was performed in triplicate for every patient. Spearman correlation analyses were performed between the six derived OCR parameters and the percentage change in platelet count from baseline (*n* = 22), with 95% confidence intervals obtained by bootstrap and correction for multiple comparisons applied using the Benjamini-Hochberg procedure. All graphs were prepared using GraphPad Prism version 8 (GraphPad Software, San Diego, USA).

Proteomic raw data were processed in Spectronaut-18 using a library-free directDIA with default cross-run normalization at the MS2 level, protein abundances were log₂-transformed in R (v4.5.1) to stabilize variance and approximate normality. Sample-wise median centering was subsequently applied by subtracting each sample’s median intensity to correct for systematic differences in total protein loading across runs. Proteins detected in fewer than 80% of replicates in all experimental groups were excluded to reduce noise from sporadic identifications. All samples were processed and acquired within a single LC-MS/MS batch; therefore, no batch correction was applied. Differential expressions between groups were assessed using two-sided Welch’s t-tests. A dual-filter strategy was applied, combining a nominal p-value < 0.05 with |log₂FC| > 0.585 (1.5-fold change), to balance sensitivity and specificity in this exploratory discovery setting. This approach was selected given the limited sample size, where formal FDR correction may excessively penalize true positives. Results were visualized using volcano plots generated with ggplot2, and dimensionality reduction was performed via PCA using the NIPALS algorithm (pcaMethods package) to accommodate missing values.

Protein–protein interaction (PPI) analysis was performed using STRING version 12.0, revealing a significant enrichment of interactions among the identified proteins (PPI enrichment *p*-value < 0.05), indicating that these associations were not due to random chance. Complementing this, KEGG analysis was carried out to identify potential pathways associated with the differentially expressed proteins (DEPs), providing insights into the biological mechanisms underlying the observed changes in gene expression. Together, these findings show that the resulting PPI network contains far more interactions than expected for a random protein set of comparable size, suggesting that the identified proteins are functionally interconnected and likely participate in related biological pathways.

## Results

### Analysis of patients’ clinical variables

All patients enrolled in the study met the predefined inclusion criteria. As shown in Table 1, no significant differences were observed between groups for most variables. A slight difference was found in sex distribution, with a lower proportion of females in the groups 2 and 3. A significant difference was observed in the following types of infection: bone and joint infection, which was more prevalent in group 3 (*p* = 0.0012), and sepsis, which was more common in group 2 (*p* = 0.0173). Significant differences were also noted in the glomerular filtration rate (*p* = 0.0239), reaching values within the optimal range in group 3. Demographic, anthropometric, and analytical variables were summarized to characterize the study population and confirm baseline comparability.

**Table 1:** Demographic, clinical, anthropometric and analytical characteristics of patients included in the study

Variables	Group 1 (*n* = 14)	Group 2 (*n* = 14)	Group 3 (*n* = 12)	*p*-value ^#^
**Demographic**				
Age (years)	74.36 ± 11.81	77.5 ± 10.04	69.44 ± 10.44	0.1779
Sex (count; %females)	5; 37.5	9; 64.3	8; 66.7	0.0837
**Clinical characteristics (count; %)**				
*Type of infection*				
Skin and soft tissue infection (cellulitis)	3; 21.43	1; 7.14	0; 0	0.1445
Bone and joint infection	3; 21.43	3; 21.43	10; 83.33	0.0012*
Urinary tract infection	2; 14.29	0; 0	0; 0	0.3311
Respiratory infection	2; 14.29	0; 0	0; 0	0.3311
Intra-abdominal infection	0; 0	2; 14.29	1; 8.33	0.3541
Pancreatitis	0; 0	0; 0	1; 8.33	0.3022
Endocarditis	0; 0	1; 7.14	0; 0	0.3858
Sepsis	4; 28.57	7; 50	0; 0	0.0173*
*Other comorbidities*				
Diabetes mellitus	6; 42.86	6; 42.86	6; 50	0.9171
**Anthropometric**				
BMI (kg/m2)	29.12 ± 7.07	27.71 ± 4.26	29.34 ± 6.32	0.7465
Obesity (count, %BMI > 30 kg/m2)	4; 28.57	3; 21.43	4; 33.33	0.7889
**Analytical**				
*Haematological parameters*				
Baseline haemoglobin levels (g/dL)	11.59 ± 2.00	10.36 ± 2.08	10.44 ± 1.89	0.2098
Haemoglobin levels at sample collection (g/dL)	11.09 ± 1.61	10.39 ± 1.79	10.49 ± 1.92	0.5375
Baseline haematocrit levels (%)	35.86 ± 6.16	32.43 ± 5.77	32.82 ± 5.50	0.2529
Haematocrit levels at sample collection (%)	34.19 ± 4.76	31.09 ± 6.97	32.27 ± 5.91	0.3900
*Inflammatory markers*				
C-reactive protein (mg/dL)	10.17 ± 10.25	7.87 ± 8.14	6.98 ± 11.10	0.6921
Procalcitonin (ng/mL)	3.98 ± 6.46	2.77 ± 5.38	0.10 ± 0.13	0.1470
*Others*				
Glomerular filtration rate (mL/min/1.73 m^2^)	59.97 ± 29.36	51.58 ± 31.48	83.79 ± 26.25	0.0239*
**Pharmacological variables**				
*Drug characteristics*				
Treatment duration (days)	3.86 ± 1.23	9.43 ± 2.03	29.50 ± 14.78	< 0.0001*
*Adverse reactions*				
Baseline platelet count (platelet/µL)	232 860 ± 85 560	299 930 ± 143 730	339 920 ± 122 930	0.0815
Platelet count at sample collection (platelet/µL)	238 140 ± 111 660	222 640 ± 96 710	230 330 ± 64 730	0.9102
Percent of platelet count Ɨ (%)	2.27	-25.77	-32.24	0.0118*

All quantitative variables are expressed as mean and standard deviation unless stated otherwise

#To detect differences between groups, Chi-square test (qualitative variables) and ANOVA with Tukey’s post hoc multiple comparisons test (quantitative variables) were used. Statistically significant differences were marked with a *

Ɨ Percentage of platelet count relative to the baseline count

It is worth noting that there are differences in the reduction of platelet count (Table 1), which shows a significant association with the duration of treatment. In other words, the greatest reduction (32.24%) occurs in group 3 (*p* = 0.0118), the group with the longest treatment duration.

The review of clinical records confirmed that no enrolled patient was receiving concomitant drugs with established mitochondrial toxicity, specifically nucleoside reverse transcriptase inhibitors (NRTIs), high-dose metformin, and chloramphenicol.

### Mitochondrial function

**Fig. 1:**
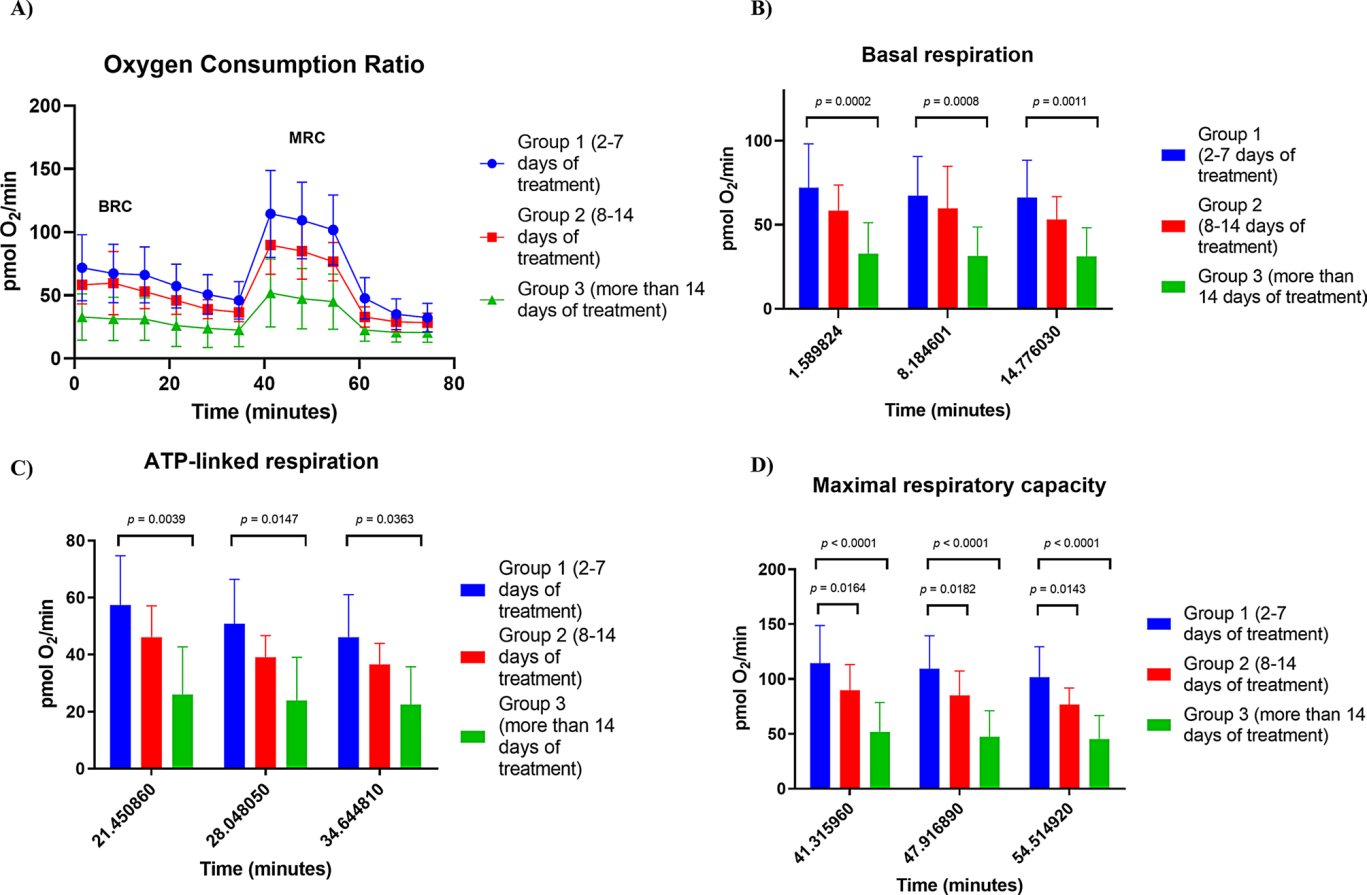
Study of mitochondrial function using the Seahorse XF Analyzer. ANOVA with Dunnett’s post hoc multiple comparisons test was performed and statistical significance was set at *p* < 0.05. **(A)** Oxygen Consumption Rate of all patients. **(B)** Fragment of basal respiration extracted from the oxygen consumption rate graph. **(C)** Fragment of ATP-linked respiration extracted from the oxygen consumption rate graph. **(D)** Fragment of maximal respiration capacity extracted from the oxygen consumption rate graph

As shown in Fig. 1A, our study noted a significant reduction in OCR in patients belonging to groups 2 and 3 with *p* < 0.0001, when compared with group 1. Focusing on the different OCR parameters, BRC exhibited significant differences between group 3 and the control group (Fig. 1B). During the intervals comprising basal respiration (1.59–14.78 min), the mean OCRs (pmol O₂/min) in patients belonging to group 1 were 71.97 ± 26.26, 67.38 ± 23.27, and 66.19 ± 22.28, respectively. In comparison, the corresponding values in group 3 were 32.90 ± 18.28, 31.34 ± 17.22, and 31.23 ± 16.94, indicating a reduction of 54.3% (*p* = 0.0002), 53.5% (*p* = 0.0008) and 52.8% (*p =* 0.0011), respectively in basal mitochondrial respiration. No statistically significant differences were observed in group 2.

In ATP-linked respiration, significant differences were also observed between group 3 and group 1 (Fig. 1C). In this case, the values for group 1, expressed in pmol O₂/min, were 57.35 ± 17.35, 50.77 ± 15.65, and 46.10 ± 14.90, whereas the corresponding values for group 3 were 26.11 ± 16.61, 23.90 ± 15.13, and 22.58 ± 13.14. These differences represent reductions in mitochondrial respiration of 54.5% (*p* = 0.039), 52.9% (*p* = 0.0147), and 51% (*p* = 0.0363), respectively. No significant differences were observed in group 2.

In turn, MRC was the parameter most strongly affected, showing a progressive decline with longer treatment durations (41.31–54.51 min). The greatest differences were detected in group 3, with statistical significance at all three MRC time points (*p* < 0.0001; Fig. 1D). In group 1, the O₂ consumption values (pmol O₂/min) at the corresponding time points were 114.42 ± 34.26, 109.27 ± 30.20, and 101.68 ± 27.62. In contrast, group 3 showed values of 51.90 ± 26.83, 47.35 ± 23.88, and 45.02 ± 21.93, corresponding to reductions of 54.6%, 56.7%, and 55.7% in MRC, respectively.

Group 2 also exhibited significant differences, though less pronounced than those in group 3. O₂ consumption values in group 2 were 89.90 ± 23.17, 85.10 ± 22.20, and 76.70 ± 15.21 pmol O₂/min. Compared with group 1 at the same time points, these represent reductions of 21.4% (*p* = 0.0164), 22.1% (*p* = 0.0182), and 24.6% (*p* = 0.0143), respectively.

These results suggest a clear temporal progression of mitochondrial toxicity. While the cell’s basic energy needs (basal/ATP) are initially preserved, the MRC is compromised early (group 2) and eventually collapses with prolonged therapy (group 3), paralleling the clinical decline in platelet counts.

As an exploratory analysis, Spearman correlations were computed between derived OCR parameters and the percentage change in platelet count from baseline (*n* = 22). Five of the six parameters showed a consistent negative directional trend, indicating that lower mitochondrial respiratory function was associated with greater platelet loss. However, after Benjamini-Hochberg correction for multiple comparisons, no parameter reached the significance threshold.

### Confocal microscopy

**Fig. 2:**
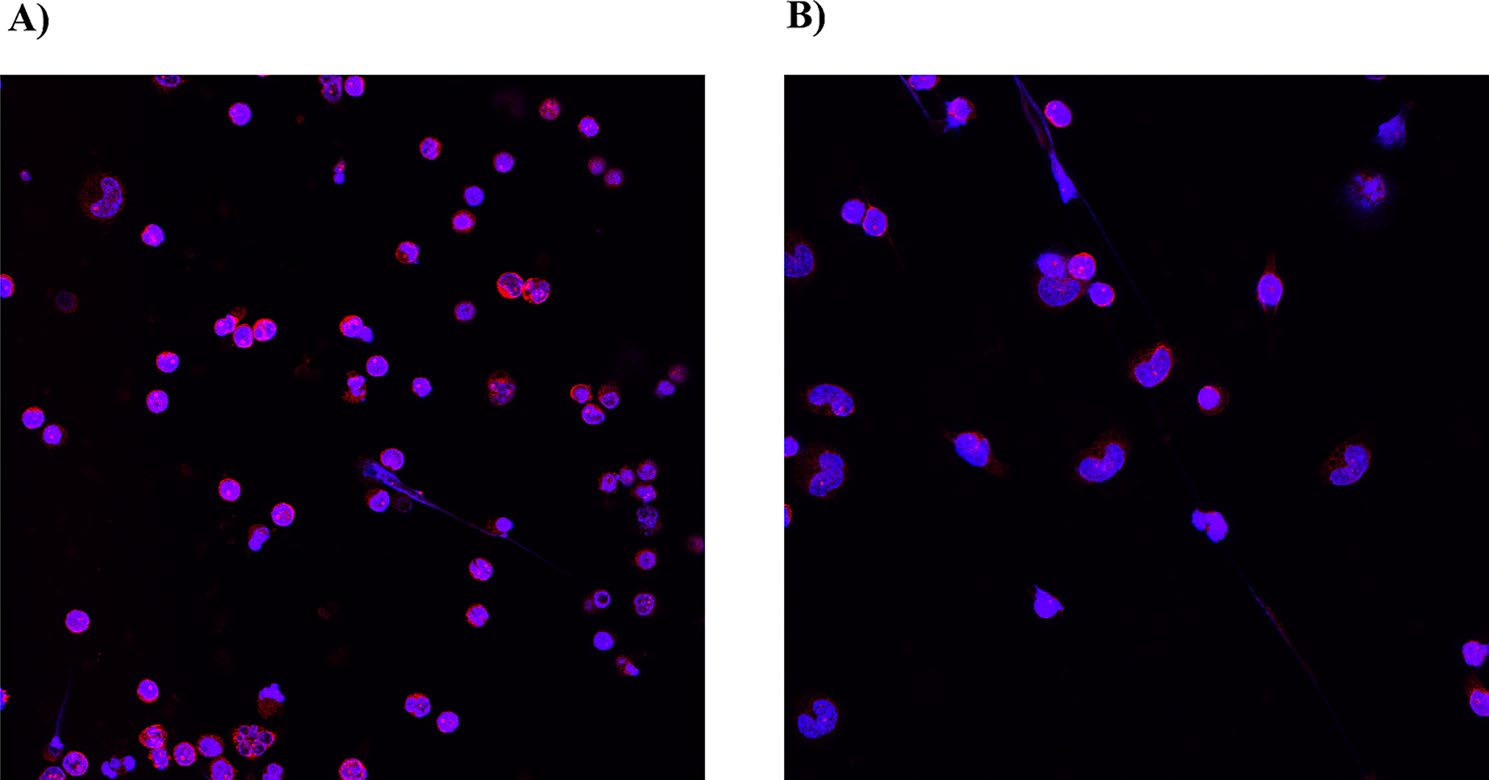
Confocal microscopy images of PBMCs isolated from patients. Images are representative of the three planes captured for each sample. **(A)** Group 1, representing 2–7 days of treatment. **(B)** Group 3, representing more than 14 days of treatment

Confocal microscopy was used to provide a qualitative visual representation of the isolated PBMCs. Based on a visual assessment of representative images, there are no major structural differences in the overall cellular morphology between patients from group 1 compared with those from group 3. However, two qualitative trends were noted. First, the images from the group 1 patients appeared to display a visually higher cell density, even though the same number of cells was initially seeded in both conditions (Fig. 2A). Furthermore, a qualitative observation of the cells isolated from group 3 patients suggested an apparent increase in the frequency of rod-shaped cells (Fig. 2B). These descriptive findings visually complement the quantitative functional data.

### Proteomic analysis

#### Protein identification

A total of 6,586 proteins were identified in PBMC samples obtained from patients. In group 2, 46 DEPs were detected, including 22 significantly downregulated and 24 significantly upregulated compared with group 1 [see Additional files 1 and 3]. In group 3, 76 proteins were found to be differentially expressed, with 33 upregulated and 43 downregulated [see Additional files 2 and 3].

Of all the downregulated proteins identified, 19 proteins were common to the groups 2 and 3 [see Additional file 4]. With respect to the upregulated proteins, a total of 21 proteins were identified as being common to both patient groups [see Additional file 4].

#### Protein-protein interaction network and KEGG enrichment analysis

**Fig. 3:**
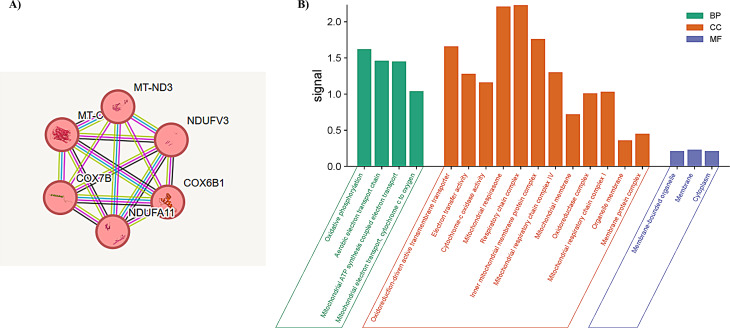
Functional analysis of the main downregulated protein cluster in Group 2 (vs. Group 1). **A)** Protein–protein interaction (PPI) network of the main cluster, retrieved from the STRING database and clustered using the MCL algorithm. Nodes represent proteins and edges represent functional and physical associations; the cluster is enriched in components of the cellular protein synthesis machinery. **(B)** Gene Ontology (GO) enrichment analysis of the cluster shown in (**A**). Strength is defined as log₁₀(observed/expected) gene counts, where higher values indicate stronger enrichment relative to the genome-wide background; bars/dots are coloured/sized by [-log₁₀ FDR / gene count]

To contextualize the observed proteomic alterations, functional enrichment analyses were conducted. If we focus on the downregulated proteins identified in group 2, the analysis delineated three distinct modules, one of which was considered functionally relevant based on interaction density and biological coherence. This cluster comprised 6 proteins implicated in respiratory electron transport (Table 2). A total of 15 interactions were identified among the 6 proteins, with a PPI enrichment *p*-value < 1.0 × 10^-16^, reflecting an exceptionally high degree of interconnectivity and indicating robust functional and biological associations (Fig. 3A).

**Table 2:** Main cluster identified among the downregulated proteins in group 2

Protein ID	Localization	Biological process	log_2_FC	FC ratio	*p* value
MT-ND3	Complex I (membrane arm, ND3)	NADH-ubiquinone oxidoreductase chain 3. Assembly of mitochondrial respiratory chain complex I [22].	-0.64	0.64	0.01
MT-CO1	Complex IV (inner membrane)	Cytochrome c oxidase subunit 1, an essential component of Complex IV [23].	-0.80	0.58	0.02
COX6B1	Complex IV	Cytochrome c oxidase subunit 6B1, which connects two COX monomers to form a dimer [24].	-0.67	0.63	0.001
COX7B	Complex IV	Indispensable for COX assembly, COX activity, and mitochondrial respiration [25]	-0.61	0.65	0.03
NDUFA11	Complex I (membrane arm, ND4)	Subunit related to sodium–proton antiporters. Assembly of mitochondrial respiratory chain complex I [26].	-0.66	0.63	0.001
NDUFV3	Complex I (peripheral arm)	Mitochondrial electron transport from NADH to ubiquinone [27].	-0.69	0.62	0.04

Gene Ontology (GO) enrichment analysis of the first protein cluster revealed strong mitochondrial involvement across Biological Process (BP), Molecular Function (MF), and Cellular Component (CC) categories. At the BP level, the most significantly enriched terms were associated with mitochondrial electron transport. Notably, specific processes such as cytochrome c to oxygen (GO:0006123), aerobic electron transport chain (GO:0019646), mitochondrial ATP synthesis coupled to electron transport (GO:0042775), OXPHOS (GO:0006119), proton motive force-driven mitochondrial ATP synthesis (GO:0042776), and mitochondrial electron transport, NADH to ubiquinone (GO:0006120) demonstrated particularly strong enrichment. Within the MF category, the most significantly enriched terms comprised cytochrome-c oxidase activity (GO:0004129), oxidoreduction-driven active transmembrane transporter activity (GO:0015453), NADH dehydrogenase (ubiquinone) activity (GO:0008137) and electron transfer activity (GO:0009055). In the CC category, the DEPs were predominantly localized to mitochondrial respiratory chain complex IV (GO:0005751), respiratory chain complex (GO:0098803), mitochondrial respirasome (GO:0005746), mitochondrial respiratory chain complex I (GO:0005747), and inner mitochondrial membrane protein complex (GO:0098800) (Fig. 3B), in agreement with the patterns observed in the BP analysis.

**Fig. 4:**
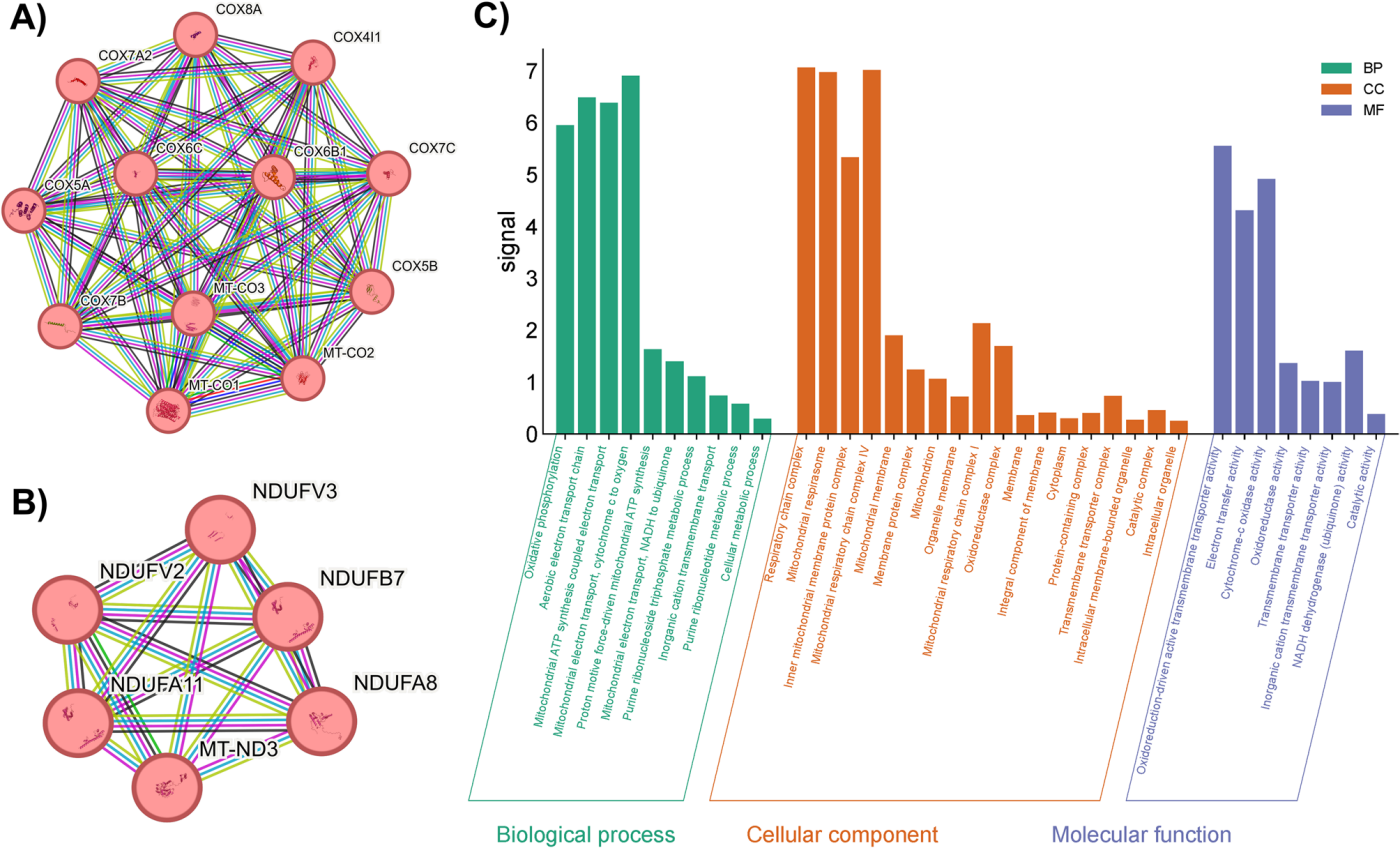
Functional analysis of the downregulated protein clusters in Group 3 (vs. Group 1. **(A**,** B)** Protein–protein interaction (PPI) networks of the two main clusters retrieved from the STRING database and clustered using the MCL algorithm. Nodes represent proteins and edges represent functional and physical associations: cluster 1 **(A)** and cluster 2 **(B)** are both enriched in components of the cellular protein synthesis machinery. **(C)** Combined GO enrichment analysis of the proteins in clusters 1 and 2. Strength is defined as log₁₀(observed/expected) gene counts; higher values indicate stronger enrichment relative to the genome-wide background

When comparing the downregulated proteins identified in the group 3, the analysis delineated three clusters again. The first was composed of 12 proteins involved in mitochondrial electron transport, specifically in cytochrome c to oxygen (BP, GO:0006123) (Fig. 4A and C; Table 3). 66 interactions were observed among these proteins, compared to the 2 interactions expected, with a PPI enrichment *p*-value < 1.0 × 10^-16^. Regarding MF, two clearly distinct groups were evident: 9 were involved in cytochrome-c oxidase activity (GO:0004129) and 10 in oxidoreductase activity (GO:0016491). All were in mitochondrial respiratory chain complex IV (CC, GO:0005751).

**Table 3:** Main clusters identified among the downregulated proteins in group 3

Cluster	Protein ID	Localization	Biological process	log_2_FC	FC ratio	*p* value
1st	MT-CO2	Complex IV	Mitochondrial-cytochrome c oxidase II, a subunit essential for the Complex IV, in promoting glutaminolysis [28].	-0.80	0.58	0.03
	MT-CO1	Complex IV (inner membrane)	Cytochrome c oxidase subunit 1, an essential component of Complex IV [23].	-1.21	0.43	0.02
	COX6B1	Complex IV	Cytochrome c oxidase subunit 6B1, which connects two COX monomers to form a dimer [24].	-1.08	0.47	0.001
	COX7B	Complex IV	Indispensable for COX assembly, COX activity, and mitochondrial respiration [25].	-0.98	0.51	0.03
	MT-CO3	Complex IV	Cytochrome c oxidase subunit 3.	-0.97	0.51	0.02
	COX6C	Complex IV	COX6C depletion leads to reduced mitochondrial fusion and impaired oxidative phosphorylation [29].	-0.63	0.65	0.04
	COX7A2	Complex IV	Structural protein bridging the association of individual complexes III and IV [30].	-0.77	0.58	0.03
	COX8A	Complex IV	COX8A is required for maintenance of the structural stability of COX monomers and dimers [31].	-0.69	0.62	0.01
	COX5A	Complex IV	Nuclear-encoded subunit of complex IV with a function in complex IV biogenesis [32].	-0.87	0.55	0.01
	COX7C	Complex IV	COX7C forms a stable contact between complexes I and IV and is required for the assembly of complex IV and the supercomplexes [33].	-0.72	0.61	0.03
	COX4I1	Complex IV	Cytochrome c oxidase subunit 4 isoform 1, involved in the early assembly stages of complex IV [34].	-0.59	0.67	0.03
	COX5B	Complex IV	Peripheral subunit of the cytochrome c oxidase complex, which contributes to the stability of this complex [35].	-0.92	0.53	0.02
2nd	MT-ND3	Complex I (membrane arm, ND3)	NADH-ubiquinone oxidoreductase chain 3. Assembly of mitochondrial respiratory chain complex I [22].	-0.64	0.64	0.01
	NDUFA8	Complex I (intermembrane surface)	NDUFA8, a subunit of complex I, enhances complex I activity, reduces mitochondrial ROS, and improves mitochondrial function [36].	-0.90	0.54	0.01
	NDUFB7	Complex I (intermembrane surface)	Accessory subunit of complex I [37].	-0.80	0.57	0.01
	NDUFV2	Complex I (peripheral arm)	Central subunit, where ubiquinone reduction occurs [38].	-0.59	0.66	0.03
	NDUFA11	Complex I (membrane arm, ND4)	Subunit related to sodium–proton antiporters. Assembly of mitochondrial respiratory chain complex I [26].	-0.66	0.63	0.001
	NDUFV3	Complex I (peripheral arm)	Mitochondrial electron transport from NADH to ubiquinone [27].	-0.69	0.62	0.04

The second cluster identified in this patient group comprised 6 proteins involved in mitochondrial ATP synthesis driven by the proton-motive force (Fig. 4B and C; Table 3). 15 interactions were revealed among these proteins, with a PPI enrichment *p*-value < 1.0 × 10^-16^. The main BP they contributed to included mitochondrial electron transport, NADH to ubiquinone (GO:0006120), with 5 of the 6 proteins participating, and complex I assembly (GO:0032981), involving 3 proteins. Regarding MF, 5 were associated with ubiquinone activity (GO:0008137), and their primary localization was in the complex I (GO:0005747), with 2 specifically situated in the mitochondrial intermembrane space (GO:0005758).

Regarding the upregulated proteins, we identified a heterogeneous group. In group 2, we obtained a single cluster consisting of three proteins, showing two interactions among them and a PPI enrichment *p*-value of 0.007. These proteins were in the inner mitochondrial membrane and were as follows: MRPL17, OXA1L and COX15. Finally, in group 3 we observed similar results. We obtained five clusters, each containing two proteins. The first one matches two of the upregulated proteins that we identified in group 3, OXA1L and COX15, with a single interaction and a PPI enrichment *p*-value of 0.022.

Collectively, the functional enrichment analysis provides a direct molecular rationale for the progressive respiratory deficits observed in the Seahorse assays. In patients treated for 8–14 days (group 2), the targeted downregulation of specific Complex IV subunits (e.g., MT-CO1, COX6B1) correlates with the early decline in maximal respiratory capacity (MRC). This suggests that the electron transport chain begins to lose its maximal energy reserve capacity before basic cellular functions are compromised.

By group 3 (treatment > 14 days), this molecular impairment broadens substantially. The profound downregulation of an extensive cluster of Complex IV proteins, coupled with a newly identified downregulation of Complex I core and accessory subunits (e.g., NDUFA8, NDUFB7, NDUFV2), is associated with a substantial impairment of key components of the respiratory chain. This means that the extensive downregulation of complex I and IV subunits directly mirrors the severe, concurrent drops in basal respiration, ATP-linked production, and MRC observed functionally. Ultimately, this molecular and functional energetic failure aligns with the onset of clinical toxicity, such as the progressive reduction in platelet counts.

## Discussion

This cross-sectional observational study evaluated mitochondrial function and the PBMC proteome in patients receiving linezolid across three treatment duration windows. The findings reveal a time-dependent association between linezolid exposure and mitochondrial dysfunction, supported by both functional and molecular evidence. However, the observational, cross-sectional design precludes causal inference, and findings should be interpreted accordingly.

The patient groups were comparable in terms of mean age, which is a direct contributor to mitochondrial dysfunction. We observed a higher proportion of women in groups 2 and 3. Haematological adverse reactions have been reported more frequently among male patients; however, this may be attributed to the primary indications (staphylococcal infections, tuberculosis, and pneumonia), which tend to be more prevalent in men. Conversely, gastrointestinal adverse reactions (nausea and vomiting) are more commonly observed in women and represent a major cause of treatment discontinuation. Consequently, these sex-related associations may be somewhat biased [2]. The groups are comparable with respect to the clinical variables assessed; significant differences are observed only in the type of infection and in GFR. In this context, group 3 showed a GFR closer to optimal ranges, which would be expected to result in more efficient linezolid clearance and lower systemic drug exposure, biasing any residual confounding towards the null and making the mitochondrial effects observed in this group potentially conservative rather than inflated. The parameters that do have an influence on mitochondrial toxicity are obesity and diabetes, and we also observe that the groups are comparable to one another [2]. A significant difference was found in the type of infection: bone and joint infection was more prevalent in group 3 (*p* = 0.0012), consistent with the longer treatment courses typically required for this condition, and therefore reflecting the nature of the clinical indication rather than an independent confounder. Nevertheless, differing infection profiles inherently carry different baseline levels of systemic inflammation, with acute presentations such as bacteremia generally associated with higher inflammatory burden than chronic bone and joint infections, which may independently influence mitochondrial function. In this regard, however, inflammatory markers (CRP and procalcitonin) did not differ significantly between groups (*p* = 0.692 and *p* = 0.147, respectively), suggesting that the net inflammatory burden at the time of sampling was broadly comparable across groups, and that this is unlikely to represent a major source of differential confounding. Additionally, although we confirmed that no enrolled patient was receiving concomitant drugs with established mitochondrial toxicity, the systematic collection of concomitant medication data was not pre-specified in the study protocol, precluding a formal analysis of potential pharmacological interactions with linezolid therapy. Given the sample sizes involved, performing fully adjusted multivariate analyses for all outcomes simultaneously was not statistically feasible and would risk overfitting, so potential confounders have therefore been addressed through descriptive comparisons, and residual confounding cannot be fully excluded.

Mild thrombocytopenia was identified in patients within the groups with the longest treatment duration. These findings agreed with the existing literature, which reported that the development of thrombocytopenia occurred more frequently in patients receiving treatment for more than 14 days [39, 40]. Consistent with this pattern, the association between OCR parameters and platelet decline followed a predominantly inverse trend, with most metrics suggesting that lower mitochondrial respiratory function is associated with greater platelet loss. Although no parameter reached significance after correction for multiple comparisons, these findings support the biological plausibility of this relationship and warrant formal evaluation in larger longitudinal cohorts.

Linezolid toxicity primarily affects organs with high energetic demand, such as the retina, peripheral nerves, and bone marrow. These tissues exhibit a high rate of aerobic metabolism and, consequently, a high mitochondrial content, which rationalizes the use of PBMCs as a surrogate model for assessing mitochondrial effects in our assays [15].

According to the endosymbiotic theory, mitochondria evolved from ancient proteobacteria, sharing ribosomal features with bacteria. This similarity makes mitochondria susceptible to ribosomal inhibitor antibiotics, which can cause clinical effects similar to primary mitochondrial disorders [5]. Mitochondrial respiration comprises several phases. On one hand, BRC, which corresponds to the OCR observed without any pharmacological modification, represents the metabolic activity required for the cell’s basal functions. BRC is followed by ATP-linked respiration, which is sensitive to oligomycin and can be used to estimate the portion of respiration dedicated to driving mitochondrial ATP synthesis [13]. In contrast, the MRC denotes the highest potential of the respiratory chain to consume oxygen and produce energy. However, BRC do not accurately reflect the capacity of cellular respiration to respond to increased energy demands; especially relevant in cell lines that temporarily encounter phases of high ATP demand. Thus, assessing the maximal capacity for substrate oxidation is highly informative for elucidating the mechanisms through which genetic modifications or pharmacological agents modulate cellular metabolism [41].

In our study, significant differences in basal and ATP-linked respirations were observed only in group 3 relative to group 1. Differences in ATP-linked respiration may also be influenced by substrate availability and oxidation, especially in the presence of mitochondrial dysfunction. In contrast, MRC values were found to be significantly reduced in both groups 2 and 3 compared with the group (1) The rate of MRC is primarily determined by substrate availability and oxidation, which depend on substrate transport across the plasma and mitochondrial membranes, as well as on key metabolic enzymes that regulate flux. Variations in this rate may also indicate changes in mitochondrial biogenesis or cristae density [13]. Collectively, these results suggest a time-dependent effect, which corresponds to the decrease in platelet count compared with baseline observed in that same group of patients. Milosevic et al. reported a reduction in BRC as well as a decrease in spare respiratory capacity (the ability of mitochondria to enhance oxidative metabolism upon increased energy demand) in cells exposed to linezolid. Moreover, this inhibitory effect persisted over time [13, 14]. Regarding spare capacity, defined as the difference between MRC and BRC, we also observed a decrease in the treatment group (2) In contrast, in the group 3, the spare capacity was not altered, as BRC and MRC were markedly reduced. Nevertheless, from a functional perspective, the cell is unable to adequately respond to ATP demands under conditions of cellular stress, since its capacity to sustain basic physiological processes and to meet maximal energy requirements is compromised. Nonetheless, when the experimental goal is simply to assess the oxidative capacity of a cell, MRC is often a more appropriate metric than spare capacity, as it reflects solely the capacity for substrate supply and oxidation [41].

On the other hand, Garrabou et al. investigated mitochondrial toxicity in PBMCs from patients after one month of linezolid therapy and they reported a 52% decline in complex IV enzymatic activity, accompanied by a 55% reduction in mitochondrial protein synthesis [5]. The mitochondrial function data obtained in our study are consistent with previously reported observations. We acknowledge that the number of samples per group (*n* = 8 for Seahorse assays and *n* = 6 for proteomics) is relatively small, which limits the overall statistical power. It is primarily constrained by the clinical challenges of prospectively recruiting complex hospitalized patients undergoing prolonged linezolid therapy, particularly for the longest-duration group (> 14 days), given that only a small fraction of the clinical population sustains therapy for this duration. Nevertheless, to our knowledge, this represents the largest patient cohort in which mitochondrial respiration has been prospectively assessed in individuals receiving linezolid therapy.

Confocal microscopy showed no morphological differences but confirmed functional disparities. PBMCs from group 1 displayed higher cell density and superoxide production, supporting the mitochondrial function assay results. The greater proportion of rod-shaped cells in group 3 likely reflects longer treatment and infection duration, stimulating increased neutrophil production. These findings are consistent with the functional data but should be considered descriptive and hypothesis-generating. Formal quantification would be required to draw definitive conclusions.

In turn, mitochondrial ATP synthesis must closely match cellular demand, and in higher organisms, this is primarily achieved through the OXPHOS system. Complex IV plays a central role by catalysing the reduction of oxygen to water, coupling ATP synthesis, and regulating energy metabolism. For this reaction 4 electrons are required and 8 of the 9 proteins identified in complex IV are involved in mediating electron transfer from cytochrome c to molecular oxygen [42]. Furthermore, multiple studies have reinforced our findings by showing a link between linezolid-induced toxicity and reduced protein synthesis in complex IV [5, 13, 15], and this connection is further supported by McKee et al., who reported that linezolid suppresses mitochondrial protein synthesis by approximately 50% at concentrations of 3 to 5 mg/L in rats and rabbits [43].

Previous literature on mitochondrial dysfunction induced by linezolid identifies complex IV of the electron transport chain as a primary target, along with several of its constituent proteins, including COX-II and CYTox I [5, 13]. Our findings corroborate these observations: three of the six proteins identified in group 2 correspond to complex IV, and in group 3 we detected a complete cluster composed of 12 complex IV proteins, including the same three detected in group 2. We additionally observed that most of the proteins identified belong to the COX family.

However, to our knowledge, this study is the first to observe an association between prolonged linezolid treatment and the downregulation of proteins localized to complex I (NADH: ubiquinone oxidoreductase) in a clinical cohort, with evidence of mitochondrial dysfunction. Prior evidence for complex I involvement has been limited to in vitro cell models and animal studies [43, 44]; our findings provide the first clinical proteomic evidence of this association. It should be noted that this evidence remains associative: a direct mechanistic link between the observed subunit downregulation and impaired complex I assembly or function would require additional targeted biochemical studies. Complex I is essential for cellular metabolism, its primary role is to oxidize NADH while reducing ubiquinone and translocating protons across the membrane. It is a large, L-shaped membrane protein complex composed of a peripheral arm (electron transfer from NADH to ubiquinone) and a membrane arm (proton translocation). It contains 14 core subunits—7 in each arm—conserved from bacteria to humans, along with approximately 30 accessory subunits found almost exclusively in the eukaryotic complex [26, 27].

The proper assembly of mitochondrial complex I requires the coordinated integration of the 44 subunits derived from two distinct genomes into its two structural domains. The hydrophobic core subunits (ND1–ND6 and ND4L), which constitute the membrane arm, are encoded by the mitochondrial genome; whereas the hydrophilic core subunits (NDUFS1, NDUFS2, NDUFS3, NDUFS7, NDUFS8, NDUFV1, and NDUFV2), forming the peripheral arm, along with all supernumerary subunits, are encoded by nuclear genes, synthesized in the cytoplasm, and subsequently imported into the mitochondria [45, 46].

The downregulated proteins belonging to complex I were distributed across both domains. As with complex IV, the three proteins identified in group 2 are likewise shared with group 3. It should be noted that the functions of several of these proteins remain incompletely characterized; however, others are known to play essential roles in the proper functioning and assembly of complex I. For instance, MT-ND3, NDUFA11 and NDUFB7 play critical roles in the complex I assembly. NDUFA11 is a factor involved in the assembly of complex I, and in vitro studies have reported that loss of this gene leads to the absence of complex I expression. Mutations in this gene are associated with disorders such as fatal infantile lactic acidaemia [27]. NDUFB7, in turn, contributes to membrane stabilization [47]. NDUFA8 is directly associated with the activity of complex I. NDUFV2 and NDUFV3 function as electron donors to the complex, providing the NADH substrate; however, NDUFV3 is not essential, as reduced expression of this factor has only a minimal impact on overall enzymatic activity [27]. Therefore, mutations that disrupt or impair complex I function generally have broader effects on overall mitochondrial activity, as observed in the mitochondrial function studies.

We acknowledge that the use of nominal p-values without formal FDR correction represents a limitation of this study. The identified differentially expressed proteins should be considered as discovery-phase candidates requiring independent validation.

Prolonged administration of linezolid has been associated not only with thrombocytopenia but also with hyperlactatemia and optic or peripheral neuropathy. These toxic manifestations closely resemble the biochemical profile of Leber’s hereditary optic neuropathy and MELAS-like syndromes (mitochondrial disorders that mimic mitochondrial encephalopathy, lactic acidosis, and stroke-like episodes), both of which result from mutations affecting mitochondrial complex I and lead to defective assembly of the enzyme complex [48, 49].

Interestingly, there is a notable similarity between the mechanisms underlying linezolid toxicity and those of chloramphenicol. The latter also impairs mitochondrial function and additionally causes bone marrow aplasia and suppression, which has largely restricted its clinical use to topical applications for ocular infections. In cells exposed to chloramphenicol, mitochondrial protein synthesis—particularly of complex I subunits—is reduced [43, 44].

In line with these observations, our study revealed a comparable deficit in the expression of complex I proteins following linezolid exposure. These findings suggest that the shared impairment of mitochondrial protein synthesis, potentially involving complex I alongside complex IV, may represent a common underlying mechanism contributing to the toxicity observed with both drugs.

Taken together, complex IV has been established as the primary mitochondrial target in prior clinical studies, based on enzymatic activity measurements in small patient cohorts [5, 12, 15]. Our study extends this evidence in three specific ways. First, we provide functional confirmation of complex IV involvement using Seahorse-based respirometry in a larger patient cohort. Second, we observed that this impairment is progressive and treatment duration-dependent, with MRC affected from 8 to 14 days and basal respiration only significantly reduced beyond 14 days. Third, we report proteomic evidence of complex I subunit downregulation in human patients receiving linezolid, while prior evidence was limited to in vitro and animal models.

This mechanistic understanding also points toward potential therapeutic rescue strategies. Recent studies suggest that exogenous ATP or antioxidant molecules like pyridoxine (vitamin B6) can mitigate linezolid-induced oxidative stress and hematological damage. Other mitochondria-targeted approaches, such as thiamine or coenzyme Q10 supplementation, could also be explored to protect energy-dependent tissues like the bone marrow during prolonged therapy [50, 51].

The detection of certain overexpressed proteins of the mitochondrial inner membrane may reflect compensatory cellular mechanisms. Nevertheless, this observation should be considered anecdotal, given the limited number of proteins identified and the heterogeneity within the groups.

Regarding pre-analytical variables, the 48-hour processing window was necessitated by clinical logistics, and cryopreservation was chosen to enable simultaneous batch analysis of all samples, minimizing inter-assay variability; both protocols were validated through internal stability testing. A fixed seeding density of 1 × 10⁶ cells per well was applied uniformly across all samples, ensuring comparable OCR measurements between groups. Although these factors could introduce minor variations in absolute respiratory measurements, their systematic application across all samples minimizes differential bias between groups.

## Conclusions

This study provides novel insights into the molecular mechanisms underlying linezolid-induced mitochondrial toxicity in patients undergoing prolonged therapy. Functional analyses showed progressively lower mitochondrial respiratory capacity with increasing treatment duration, particularly in patients treated for more than 14 days. This pattern was accompanied by reductions in platelet counts, supporting a relationship between longer exposure to linezolid and mitochondrial and hematological alterations.

Proteomic profiling identified a significant downregulation of proteins involved in mitochondrial ATP synthesis, primarily those associated with complexes I and IV of the electron transport chain. Collectively, these findings extend current understanding of linezolid-induced mitochondrial dysfunction and suggest that complex I proteins may also be involved, in addition to the previously described impairment of complex IV.

Finally, these results should be interpreted as hypothesis-generating and may help guide future studies aimed at clarifying the mechanisms of linezolid-related toxicity and exploring potential strategies to prevent or mitigate mitochondrial dysfunction during prolonged treatments.

## Supplementary Information

Below is the link to the electronic supplementary material.

Supplementary Material 1: Additional file 1: Downregulated proteins identified in group 2 compared with group 1. Table detailing the downregulated proteins identified in group 2 compared with group 1, including average log2, log2 fold-change, fold-change ratio, and p-values.

Supplementary Material 2: Additional file 2: Downregulated proteins identified in group 3 compared with group 1. Table detailing the downregulated proteins identified in group 3 compared with group 1, including average log2, log2 fold-change, fold-change ratio, and p-values.

Supplementary Material 3: Additional file 3: Volcano plot of differentially abundant proteins between treatment-duration group. Each point represents a single protein; the x-axis displays the log2fold change and the y-axis the -log10 p-value. Proteins meeting the selection criteria (|log₂FC| ≥ 0.58 and *p-value* < 0.05) are highlighted: downregulated proteins are shown in blue and upregulated proteins in red; non-significant proteins are shown in grey. (A) Group 1 (2–7 days of treatment) vs. group 2 (8–14 days of treatment). (B) Group 1 (2–7 days of treatment) vs. group 3 (> 14 days of treatment).

Supplementary Material 4: Additional file 4: Venn diagrams of differentially abundant proteins shared between groups. Each circle represents the set of differentially abundant proteins identified in one patient group compared with group 1; overlapping regions correspond to proteins that are common to multiple groups, and the non-overlapping regions show proteins that are unique to a single group. (A) Downregulated proteins identified in groups 2 vs. group 1 and group 3 vs. group 1. (B) Upregulated proteins identified in groups 2 vs. group 1 and group 3 vs. group 1.

## Data Availability

The original contributions presented in this study are included in the article and its Additional Files. Further inquiries or requests for datasets can be directed to the corresponding authors.
