# Supplementary figures and images for: Prolonged linezolid therapy induces progressive mitochondrial dysfunction in human peripheral blood mononuclear cells
Source: J Transl Med. 2026 Jun 9;24:789. doi: 10.1186/s12967-026-08406-5 (PMC13281431; doi:10.1186/s12967-026-08406-5)

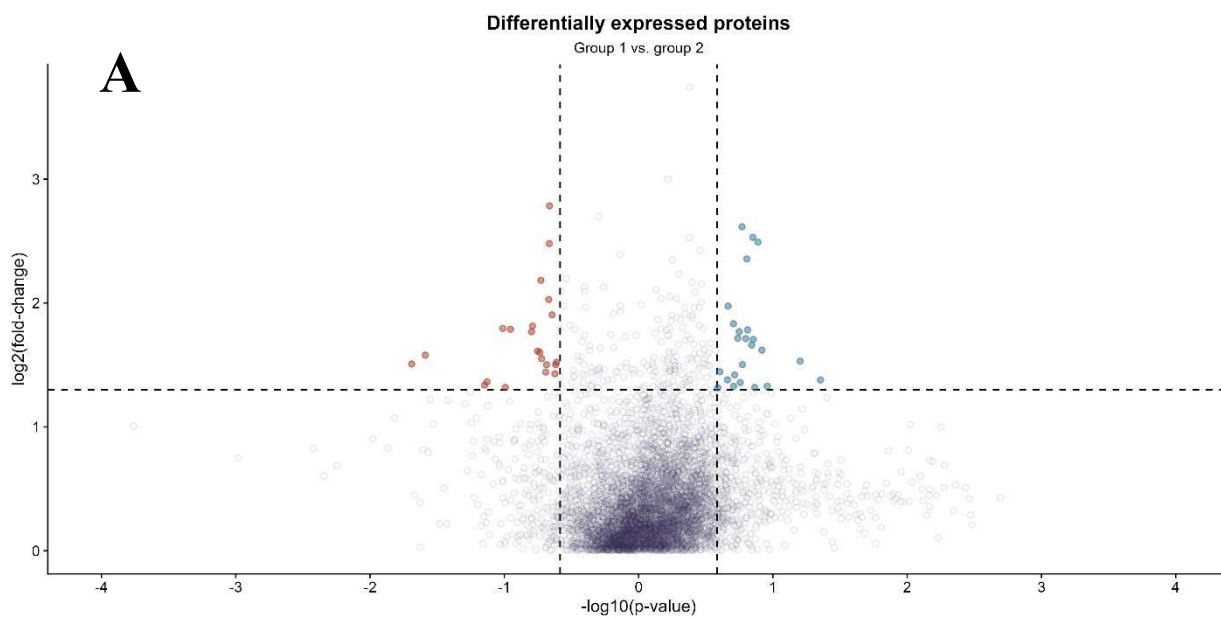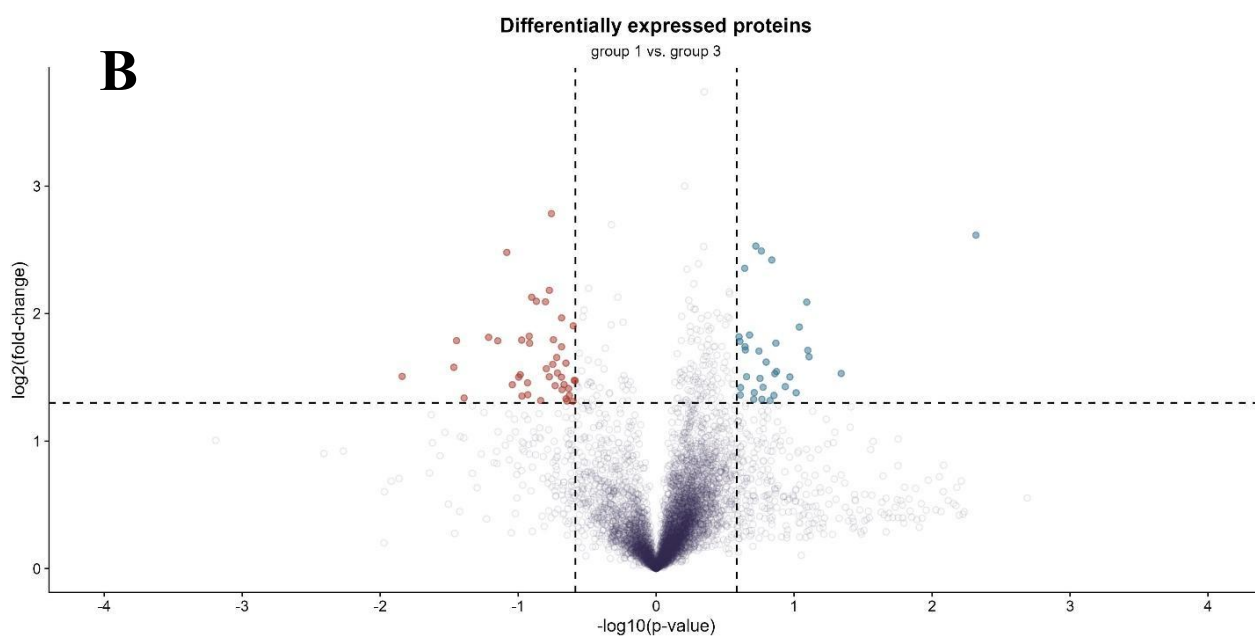

Supplement: Supplementary file 3 — Supplementary Material 3: Additional file 3: Volcano plot of differentially abundant proteins between treatment-duration group. Each point represents a single protein; the x-axis displays the log2fold change and the y-axis the -log10 p-value. Proteins meeting the selection criteria (|log₂FC| ≥ 0.58 and p-value < 0.05) are highlighted: downregulated proteins are shown in blue and upregulated proteins in red; non-significant proteins are shown in grey. (A) Group 1 (2–7 days of treatment) vs. group 2 (8–14 days of treatment). (B) Group 1 (2–7 days of treatment) vs. group 3 (> 14 days of treatment). [file 12967_2026_8406_MOESM3_ESM.pdf]

**A**

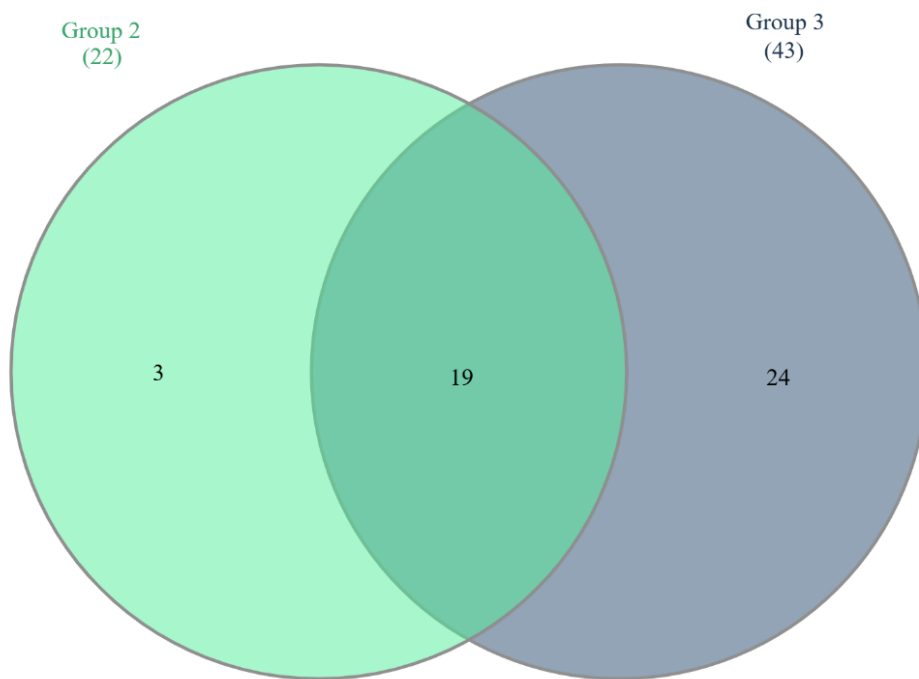

**B**

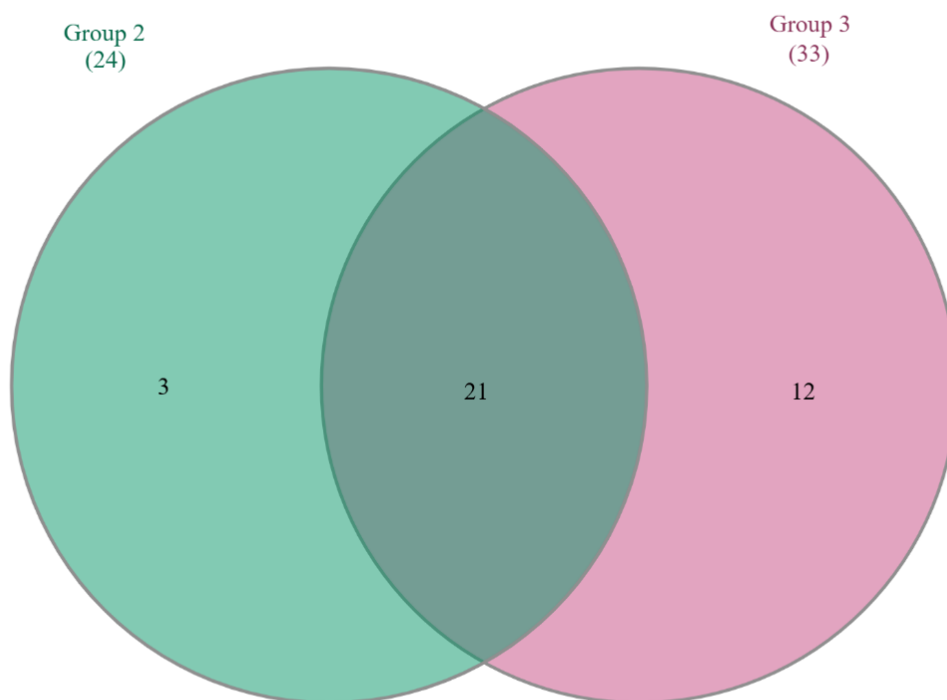

Supplement: Supplementary file 4 — Supplementary Material 4: Additional file 4: Venn diagrams of differentially abundant proteins shared between groups. Each circle represents the set of differentially abundant proteins identified in one patient group compared with group 1; overlapping regions correspond to proteins that are common to multiple groups, and the non-overlapping regions show proteins that are unique to a single group. (A) Downregulated proteins identified in groups 2 vs. group 1 and group 3 vs. group 1. (B) Upregulated proteins identified in groups 2 vs. group 1 and group 3 vs. group 1. [file 12967_2026_8406_MOESM4_ESM.pdf]
